# Generating homozygous mutant populations of barley microspores by ethyl methanesulfonate treatment

**DOI:** 10.1007/s42994-023-00108-6

**Published:** 2023-06-28

**Authors:** Linli Huang, Guangqi Gao, Congcong Jiang, Guimei Guo, Qiang He, Yingjie Zong, Chenghong Liu, Ping Yang

**Affiliations:** 1 Biotech Research Institute, Shanghai Academy of Agricultural Sciences/Shanghai Key Laboratory of Agricultural Genetics and Breeding, Shanghai, 201106 China; 2 Institute of Crop Sciences, Chinese Academy of Agricultural Sciences, Beijing, 100081 China

**Keywords:** Barley, Mutagenesis, Microspore culture, Double-haploid (DH), Homozygous mutant

## Abstract

Induced mutations are important for genetic research and breeding. Mutations induced by physical or chemical mutagenesis are usually heterozygous during the early generations. However, mutations must be fixed prior to phenotyping or field trials, which requires additional rounds of self-pollination. Microspore culture is an effective method to produce double-haploid (DH) plants that are fixed homozygotes. In this study, we conducted ethyl methanesulfonate (EMS)-induced mutagenesis of microspore cultures of barley (*Hordeum vulgare*) cultivar ‘Hua30’ and landrace ‘HTX’. The EMS concentrations were negatively correlated with the efficiency of callus induction and the frequency of mutant plant regeneration. The two genotypes showed different regeneration efficiencies. The phenotypic variation of the regenerated M_1_ plants and the presence of genome-wide nucleotide mutations, revealed by whole-genome sequencing, highlight the utility of EMS-induced mutagenesis of isolated microspore cultures for developing DH mutants. Genome-wide analysis of the mutation frequency in the regenerated plants revealed that a considerable proportion of mutations resulted from microspore culture (somaclonal variation) rather than EMS-induced mutagenesis. In addition to producing a population of 1972 homozygous mutant lines that are available for future field trials, this study lays the foundation for optimizing the regeneration efficiency of DH plants and the richness of mutations (mainly by fine-tuning the mutagen dosage).

## Introduction

Mutation-based breeding accelerates crop improvement by generating new genetic diversity beyond natural variation (Jankowicz-Cieslak et al. 2017). The Mutant Variety Database (MVD), which is maintained by the Joint Food and Agriculture Organization of the United Nations (FAO) and the International Atomic Energy Agency (IAEA) Centre of Nuclear Techniques in Food and Agriculture (https://nucleus.iaea.org/sites/mvd/), lists over 3000 mutant varieties from a range of plant species, including cereal (staple food) crops, economically important crops (e.g., soybean [*Glycine max*], cotton [*Gossypium hirsutum*], rapeseed [*Brassica napus*]), and horticultural species. The generation of mutagenized populations with uniform genetic backgrounds but enriched with genetic/phenotypic variation has greatly accelerated the modern genetics research using forward and reverse genetic approaches (Uauy et al. 2017; Jiang et al. 2022).

Radiation methods, such as X-ray and fast-neutron treatment, were commonly used for mutagenesis during the first three-quarters of the last century (Lundqvist 2014). These physical agents induce breaks in double-stranded DNA and the deletion of chromosomal segments. The iconic barley (*Hordeum vulgare*) cultivar (*cv*.) ‘Golden Promise’, which is the mainstay of the European brewing industry and is widely employed as the receptor genotype during barley transformation, was developed by γ-ray-induced mutation of the historical *cv*. ‘Maythorpe’, followed by selection for semi-dwarf, sturdy culms, and higher yield performance (Schreiber et al. 2020). In the recently released barley pan-genome database (Jayakodi et al. 2020), Golden Promise has the smallest genome and the fewest genes among representative cultivars/accessions, possibly due to the γ-ray-induced segmental deletions in its genome.

Since the 1970s, chemical mutagens have become increasingly popular compared to radiation (Lundqvist 2014). Commonly used chemical mutagens include sodium azide (NaN_3_), *N*-methyl-*N*-nitrosourea (MNU), and ethyl methanesulfonate (EMS), each of which induces nucleotide transitions (predominantly G–A, or C–T). Several chemically induced mutant populations have been developed in major crops and other economically important plants (Abe et al. 2012; Henry et al. 2014; Jiao et al. 2016; Gupta et al. 2017; Li et al. 2017; Schreiber et al. 2019; Gao et al. 2020; Sashidhar et al. 2020; Nie et al. 2021). Mutants in an elite parental cultivar/variety background with desirable performance for a specific trait (e.g., salt tolerance) can readily be used to develop new varieties via genetic improvement (Takagi et al. 2015).

Double haploid (DH) technology rapidly generates completely homozygous lines, which greatly shortens the duration of breeding (Ren et al. 2017; Ma et al. 2018). DHs are commonly produced by androgenesis, a highly efficient method involving the culture of isolated microspores (Esteves et al. 2014). In this technique, microspores are isolated from anthers prior to culture. The isolated microspores can be diverted from the normal gametophytic developmental pathway to a sporophytic pathway, subsequently producing embryogenic calli and haploid or DH plants (Lu et al. 2008; Ferrie and Caswell 2011). This procedure can be accelerated by optimizing the culture conditions, such as introducing nutrients into the culture medium (Shrivastava et al. 2021). An isolated microspore culture provides a large number of single haploid cells that can be subjected to uniform treatments. Thousands of embryogenic calli can thus be rapidly induced, many of which can later develop into regenerated plants (Li and Devaux 2005; Esteves et al. 2014). In recent years, higher frequencies of embryogenic callus formation and green plantlet regeneration have been achieved in several plant species, such as rice (*Oryza sativa*), wheat (*Triticum aestivum*), tobacco (*Nicotiana tabacum*), rapeseed, and eggplant (*Solanum melongena*) (Shariatpanahi et al. 2006; Mohammadi et al. 2012; Shariatpanahi and Ahmadi 2016; Dong et al. 2021; Calabuig-Serna et al. 2022; Rahman et al. 2022). An efficient isolated microspore culture system was established in barley (*Hordeum vulgare*) following the optimization of factors that affect microspore embryogenesis (Lu et al. 2008, 2016).

The heterozygosity of early-generation plant populations produced by chemically induced mutagenesis, via seed treatment, makes it difficult to identify phenotypic variation in quantitative or recessive traits. EMS mutagenesis of isolated microspore cultures can be used to produce DH lines with fixed homozygous mutations. Moreover, microspore mutagenesis is highly efficient, as millions of microspores can be treated at the same time in a small space, such as a Petri dish. This strategy is practical, as many examples of phenotypic variation have been observed in DH lines derived from mutagenesis of microspores in Ethiopian mustard (*Brassica carinata*) (Barro et al. 2001), Chinese cabbage (*Brassica rapa* spp. *pekinensis*) (Huang et al. 2016), and barley (Gao et al. 2018). In the current study, we developed a homozygous mutant population of cultivated barley by EMS-induced mutagenesis of microspores from two genotypes. We evaluated the effects of plant genotype and EMS dosage as well as the mutation efficiency, via whole-genome sequencing. Our findings highlight the efficiency of our technique for producing homozygous mutant populations in barley.

## Materials and methods

### Plant materials and growth conditions

Two barley genotypes were included in this study: the cultivar ‘Hua30’, which was developed by anther culture from a cross between the spring two-rowed *cv*. ‘Xiumai 1’ and the breeding line ‘82,164’; and the landrace ‘HTX’ (Jiang et al. 2022). The seeds were sown in the field at Shanghai Academy of Agricultural Science (Shanghai, China) in November 2018, and spikes were collected in March 2019 for isolated microspore culture. Green plantlets at the 4- to 5-leaf stage regenerated from microspore culture were grown for seed multiplication under normal greenhouse conditions at the field station (Kunming, Yunnan province, China). Five seedlings were planted in each pot containing 25 kg of local field soil, and 30 g NPK compound fertilizer was applied to each pot. The plants were watered once or twice per week and treated with 30 g of compound fertilizer after flowering. Aphids and powdery mildew were controlled by regular spraying with the pesticides imidacloprid and carbendazim.

### Microspore culture, EMS treatment, and ploidy determination

**Fig. 1:**
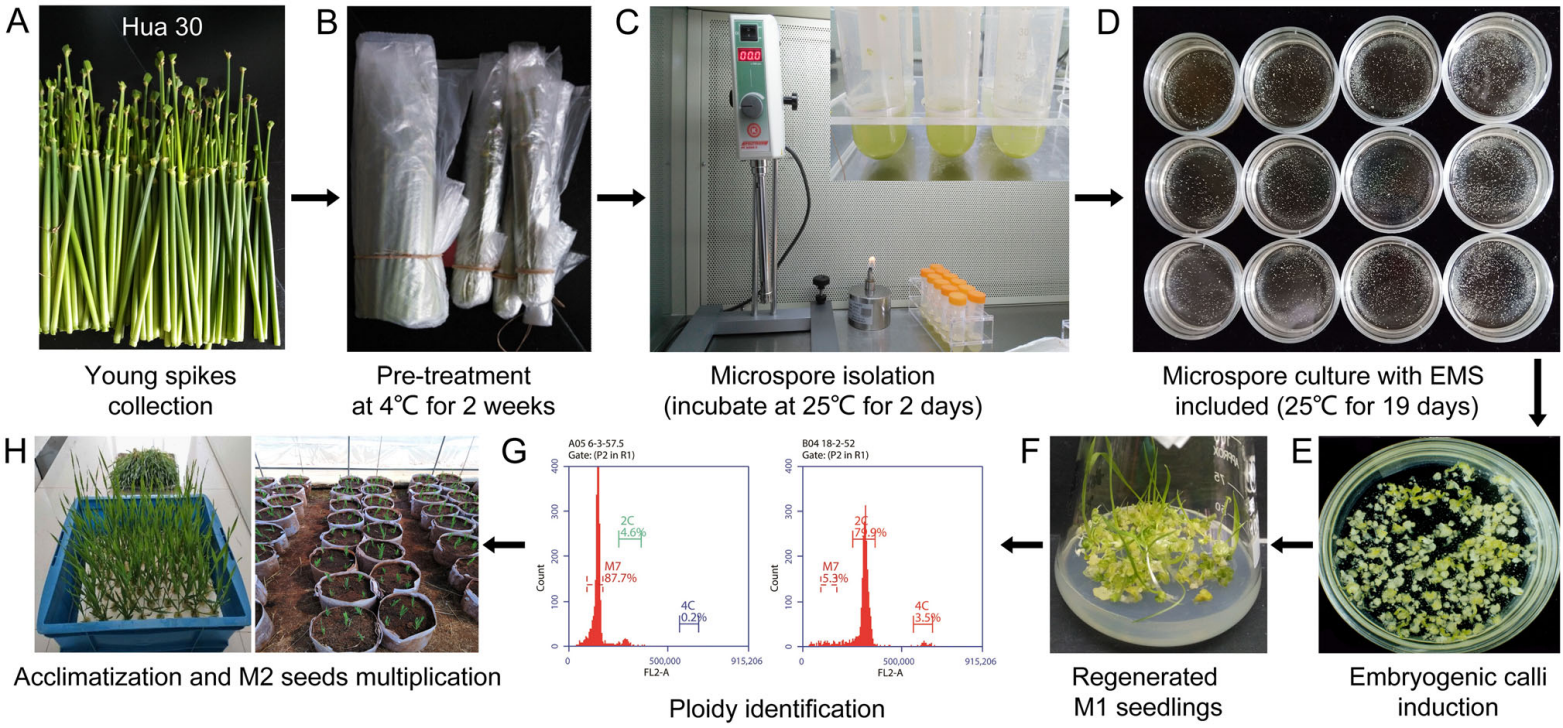
Pipeline used to produce homozygous mutants by microspore culture. Various concentrations of the mutagen EMS (0, 50, 100, 200, 300 mg/L) were included in the induction medium at the microspore culturing step. **A** Collected young spikes; **B** sample pre-treatment; **C** microspore isolation; **D** microspore culture with EMS treatment; **E** Induced embryogenic calli; **F** regenerated M_1_ plantlets; **G** typical results of ploidy determination; **H** plant cultivation and seed multiplication in the greenhouse

Microspore culture was performed, as previously described (Lu et al. 2008). Briefly, the collected spikes were subjected to cold pre-treatment at 4 °C for 2 weeks. The microspores were collected by crushing the anthers from surface-sterilized spikes and incubated in extraction buffer (pH 5.8, containing 330 mM mannitol, 10 mM CaCl_2_, and 4.6 mM MES hydrate) at 25 °C for 2 days. The microspore titer was adjusted to a density of 5.0 × 10^5^ microspores/mL for embryogenic callus induction and cultured at 25 °C for 19 days in the dark in callus induction medium alone or containing EMS at a concentration of 50, 100, 200, or 300 mg/L (Fig. 1); the callus induction medium was N6 medium (pH 5.8) supplemented with 4.5 μM 2,4-D, 2.3 μM KT, 0.7 mM hydrolyzed casein, 4.6 mM MES hydrate, 10 mM glutamine, and 250 mM maltose. Following induction, the embryogenic calli were transferred to differentiation medium (pH 5.8) composed of 2/3 Murashige and Skoog (MS) basal medium supplemented with 2.2 μM 6-BA, 7.0 μM KT, 0.25 μM NAA, and 83.3 mM maltose. The induced embryogenic calli were carefully removed from the medium by pipetting, weighed, and the regenerated plantlets, surviving plants, and albino plants counted.

The ploidy levels of a subset of regenerated green plantlets were assessed with a BD Accuri C6 flow cytometer (BD Biosciences, Franklin Lakes, NJ, USA). In brief, a 0.5–1 cm piece of a leaf blade was placed into a 1.5 mL tube, ground in 1 mL of liquid nitrogen, and re-suspended in 0.5 mL buffer solution (15 mM Tris–HCl, 2 mM Na_2_EDTA, 80 mM KCl, 20 mM NaCl, 0.1% [v/v] Triton X-100). The samples were filtered to remove debris, incubated on ice for at least 5 min, and stained by adding 0.2 mL propidium iodide (PI) in RNase staining buffer solution (BD-Pharmingen). Following incubation on ice in the dark for 30 min, the samples were analyzed on a BD Accuri C6 flow cytometer, and statistical analysis was performed using BD Accuri C6 Software. Using the position of the DNA peak from mesophyll cells of different ploidy levels in the control group as a standard, the ploidy levels of the regenerated plants were determined. Rooted green plantlets were acclimated for 1 to 2 weeks by hydroponics, transferred to soil for seed multiplication, and collected as DH lines.

### DNA extraction and whole-genome sequencing

The leaves of seedling were sampled from germinated *cv.* ‘Hua30’ seedlings and from Hua30 and HTX seedlings generated by microspore culture. Genomic DNA was extracted from the samples and its concentration quantified following a standard procedure (Yang et al. 2013). Following a quality check, the DNA samples were used for library construction, followed by sequencing using an Illumina NovaSeq platform (Novogene, Beijing) following a standard protocol (Jiang et al. 2022).

### Read mapping and identification of mutations

Raw reads were trimmed and filtered to obtain clean reads using fastp (v0.20.1) with default parameters (Chen et al. 2018). The clean reads were mapped to the assembled MorexV3 (https://doi.org/10.5447/ipk/2021/3) reference genome (Mascher et al. 2021) with bwa (v0.7.17) (Li and Durbin 2009). The mapped reads were sorted using samtools (v1.10) (Li et al. 2009), and the duplicates generated by PCR were marked and removed using the MarkDuplicates function of GATK4 (v4.2.6.1) (McKenna et al. 2010). Only reads with a mapping quality ≥ 20 were subjected to variant calling using BCFtools (v1.9) (Li 2011).

First, single-nucleotide polymorphisms (SNPs) in each sample of germinated or regenerated plants were called against the MorexV3 reference genome and subjected to the following filtration steps: (1) only bi-allelic variants were accepted; (2) genotype calls were considered valid when their read depth was ≥ 2 and ≤ 30 for each sample; (3) heterozygous SNPs were not considered. The number of mutation sites in each regenerated plant sample was then calculated as the number of mutation sites in the SNP matrix for each sample against MorexV3 compared to those in the SNP matrix against MorexV3 in plants of the corresponding genotype germinated from seeds. Only SNPs meeting the following criteria were accepted: (i) calls for HTX or Hua30 were homozygous (genotype call 0/0 or 1/1 in VCF format); (ii) the genotype calls in the mutant samples were homozygous and different from the HTX or Hua30 genotype calls; and (iii) the alternate allele was only present in a single sample. The mutation frequency for each mutant was calculated as the number of SNPs divided by the cumulative size of the whole genome sequence or gene-coding region.

## Results

### Different genotypes show different rates of plant regeneration following microspore mutagenesis

**Fig. 2:**
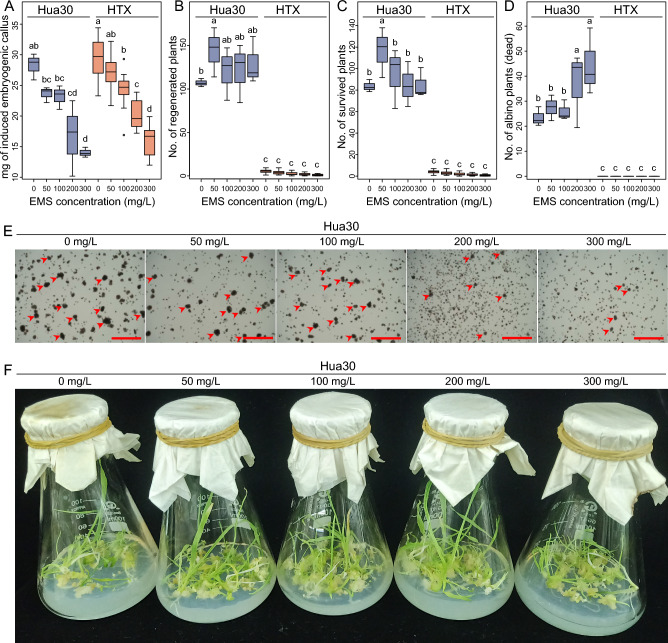
The effects of mutagen dosage and genotype on the embryogenesis and regeneration of microspore cultures. Various traits were quantified: weight of induced embryogenic calli on each plate (**A**), number of regenerated surviving plantlets (**B**), number of surviving regenerated green plantlets (**C**), and number of albino plantlets that eventually died (**D**). The number of regenerated plantlets on each plate of embryogenic callus induction medium was recorded, and three plates were quantified per treatment. Statistical analysis was conducted using Tukey’s Honest Significant Difference (*P* = 0.05) with three independent replicates. **E** Induced embryogenic calli treated with different EMS concentrations. Arrows represent microspore-derived embryogenic calli. Scale bars, 100 μm. **F** Regenerated plantlets from microspores treated with different concentrations of EMS. Two and five independent experiments were conducted for Hua30 and HTX, respectively; one experiment per genotype is shown in the figure

We subjected the two barley genotypes Hua30 and HTX to EMS-induced mutagenesis of isolated microspore cultures and investigated the rates of embryogenic callus induction and mutagenized plant regeneration. There was no significant difference in the rate of embryogenic callus induction between Hua30 and HTX at the same EMS concentration (Fig. 2A). However, the number of regenerated plants, surviving plants, and albino plants was significantly lower for HTX than Hua30, regardless of EMS concentration (Fig. 2B–D). Indeed, the number of surviving HTX plants was less than 10% that of Hua30 (Fig. 2C). These results demonstrate that the genotypes Hua30 and HTX show little difference in embryogenic callus induction but large differences in plant regeneration following microspore mutagenesis.

### EMS concentration is negatively correlated with embryogenic callus induction and plant regeneration

We examined whether EMS treatment had dose-dependent effects on embryogenic callus induction or plant regeneration. We observed a significant reduction in callus yield with increasing EMS concentration (Fig. 2E). Compared to the control (without EMS treatment), the number and the size of calli decreased with increasing EMS concentration. When the EMS concentration increased to 300 mg/L, we observed about a 50% reduction in the yield of embryogenic calli in both Hua30 and HTX (Fig. 2A). Thus, treatment with higher concentrations of EMS significantly impairs callus induction.

In Hua30, the number of regenerated plants gradually decreased with increasing EMS concentration (Fig. 2B and F). We obtained the greatest number of surviving Hua30 plants at an EMS concentration of 50 mg/L, and the fewest at an EMS concentration of 300 mg/L (Fig. 2C). Accordingly, the number of albino plants in Hua30 significantly increased when using higher concentrations of EMS (Fig. 2D). For HTX, which had a significantly lower ratio of regenerated plants compared to Hua30, different EMS concentrations had little effect on the number of regenerated plants, surviving plants, or albino plants.

### Generation of an EMS-induced homozygous mutant population

**Fig. 3:**
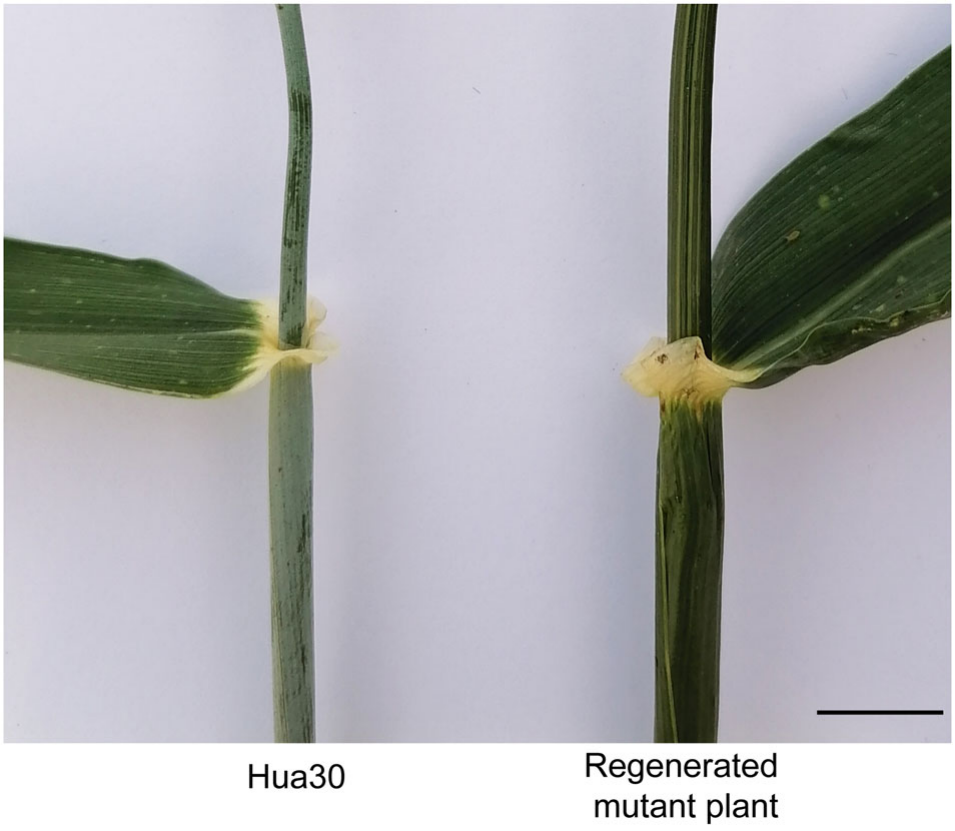
A mutant with a wax-less (non-glaucous) phenotype on the uppermost internode and flag leaf sheath observed among the regenerated double haploid M_1_ population of *cv*. ‘Hua30’. Scale bar, 1 cm

We self-pollinated the surviving plants (M_1_) to produce seeds from the DH homozygous mutants. Of the 2793 regenerated plants, derived from seven isolated microspore culture experiments, 1972 M_2_ DH lines (70.64%) were fertile and produced seeds (Table 1). These M_2_ lines included 99 DH HTX lines and 1873 DH Hua30 lines. For Hua30, we collected seeds from 1818 DH lines, comprising 258, 434, 688, and 438 lines that were treated with EMS concentrations of 50 mg/L, 100 mg/L, 200 mg/L, or 300 mg/L, respectively. We noticed one regenerated M_1_ plant lacking a wax coating (non-glaucous) on the uppermost internode and flag leaf sheath (Fig. 3). This phenotypic variation suggests that EMS treatment of isolated microspores induces mutations in the barley genome.

**Table 1:** Viability and fertility of double-haploid plants derived from microspore cultures treated with different concentrations of EMS

Genotype	EMS concentration (mg/L)	Number of DH lines	Number of harvested lines	% of lines harvested
HTX	0	45	34	76
HTX	50	32	22	69
HTX	100	27	12	44
HTX	200	36	19	53
HTX	300	24	12	50
Hua30	0	94*	55	59
Hua30	50	481*	258	54
Hua30	100	605	434	72
Hua30	200	787	688	87
Hua30	300	662	438	66

*The number of DH lines does not represent all regenerated plantlets from isolated microspore culture, since a subset of these was discarded prior to seed multiplication due to the predicted lower mutation frequencies. The number of DH lines, harvested lines, and fertile plants was derived from two experiments for Hua30 and five experiments for HTX

### Examining mutation frequency by whole-genome sequencing

To evaluate the mutation frequencies in the homozygous mutant populations, we conducted whole-genome sequencing via the Illumina approach on a subset of plants regenerated from microspore culture. We obtained 25.15–47.58 Gb of clean data, representing approximately 6–11 × coverage of the barley reference genome (Morex V3, 4.2 Gb) (Mascher et al. 2021), for each Hua30 and HTX sample (Table 2).

**Table 2:** Single-nucleotide polymorphisms revealed by whole-genome sequencing

Sample ID	Genotype	EMS treatment		Clean bases (Gb)	Genome mapped^a^	Genome-wide		Gene Region	
		Tissue	mg/L			Homozygous mutations	Ratio/Mb^b^	Homozygous mutations	Ratio/Mb^b^
HTX-2.8_1	HTX	Seed^c^	2800	42.94	3.58	15,728	4.40	323	2.61
HTX-2.8_2	HTX	Seed^c^	2800	46.47	3.60	13,936	3.87	314	2.51
HTX-2.8_3	HTX	Seed^c^	2800	48.49	3.61	13,355	3.70	319	2.55
HTX-4_1	HTX	Seed^c^	4000	45.40	3.60	25,991	7.23	781	6.26
HTX-4_2	HTX	Seed^c^	4000	48.42	3.64	13,769	3.79	312	2.46
HTX-4_3	HTX	Seed^c^	4000	49.00	3.62	9100	2.51	230	1.82
HTX-0_1	HTX	Microspore	0	38.88	3.56	3987	1.12	94	0.76
HTX-0_2	HTX	Microspore	0	32.41	3.51	4269	1.22	85	0.71
HTX-0_3	HTX	Microspore	0	32.40	3.47	4636	1.34	90	0.75
HTX-0.3_1	HTX	Microspore	300	25.97	3.41	5359	1.57	164	1.42
HTX-0.3_2	HTX	Microspore	300	27.64	3.42	5894	1.72	143	1.23
HTX-0.3_3	HTX	Microspore	300	31.14	3.49	4967	1.42	107	0.90
Hua-0_1	Hua30	Microspore	0	47.58	3.57	6641	1.86	147	1.19
Hua-0_2	Hua30	Microspore	0	44.75	3.57	6228	1.75	132	1.07
Hua-0_3	Hua30	Microspore	0	36.70	3.50	6690	1.91	174	1.46
Hua-0.3_1	Hua30	Microspore	300	30.75	3.52	8186	2.32	227	1.89
Hua-0.3_2	Hua30	Microspore	300	27.02	3.48	7129	2.05	191	1.61
Hua-0.3_3	Hua30	Microspore	300	25.15	3.45	7555	2.19	180	1.56

^a^The reference genome MorexV3 (Mascher et al. 2021) was used to map reads and for variant calling. The mapped genomic regions were calculated using the mapped reads filtered with Q20

^b^The ratio of homozygous mutations per sample was calculated as the number of homozygous SNPs divided by the cumulative size of the genomic regions mapped with high-quality reads

^c^These datasets were obtained from an earlier publication (Jiang et al. 2022) and subjected to read mapping and variant calling

To determine whether the mutations were induced by microspore culture (control condition, 0 mg/L) or by microspore culture in the presence of EMS (300 mg/L), we analyzed the mutation sites in the regenerated Hua30 and HTX plants. We detected a higher genome-wide mutation frequency in Hua30 than in HTX, regardless of treatment (Table 2). We detected an average of 1.84 and 1.23 mutations/Mb in Hua30 and HTX seedlings regenerated from seedlings derived from microspore culture on growth medium lacking EMS. A treatment with 300 mg/L EMS in the induction medium resulted in a significant increase in mutation frequency (2.19 mutations/Mb in Hua30, *P* < 0.01; 1.57 mutations/Mb in HTX, *P* < 0.05) compared to EMS-free medium. After subtracting the number of mutations generated by microspore culture, there were approximately 0.35 and 0.34 EMS-induced mutations/Mb in regenerated Hua30 and HTX seedlings, respectively. Specifically, for the gene-coding regions, we observed a similar pattern of induced mutations (Table 2). We also examined whole-genome sequencing datasets from seeds subjected to EMS-induced mutagenesis (Jiang et al. 2022) to compare the mutation frequencies obtained using different treatment approaches (seeds *vs.* isolated microspores). We determined that HTX seeds treated with 2800 mg/L and 4000 mg/L EMS accumulated 3.99 mutations/Mb and 4.51 mutations/Mb, respectively (Table 2).

**Fig. 4:**
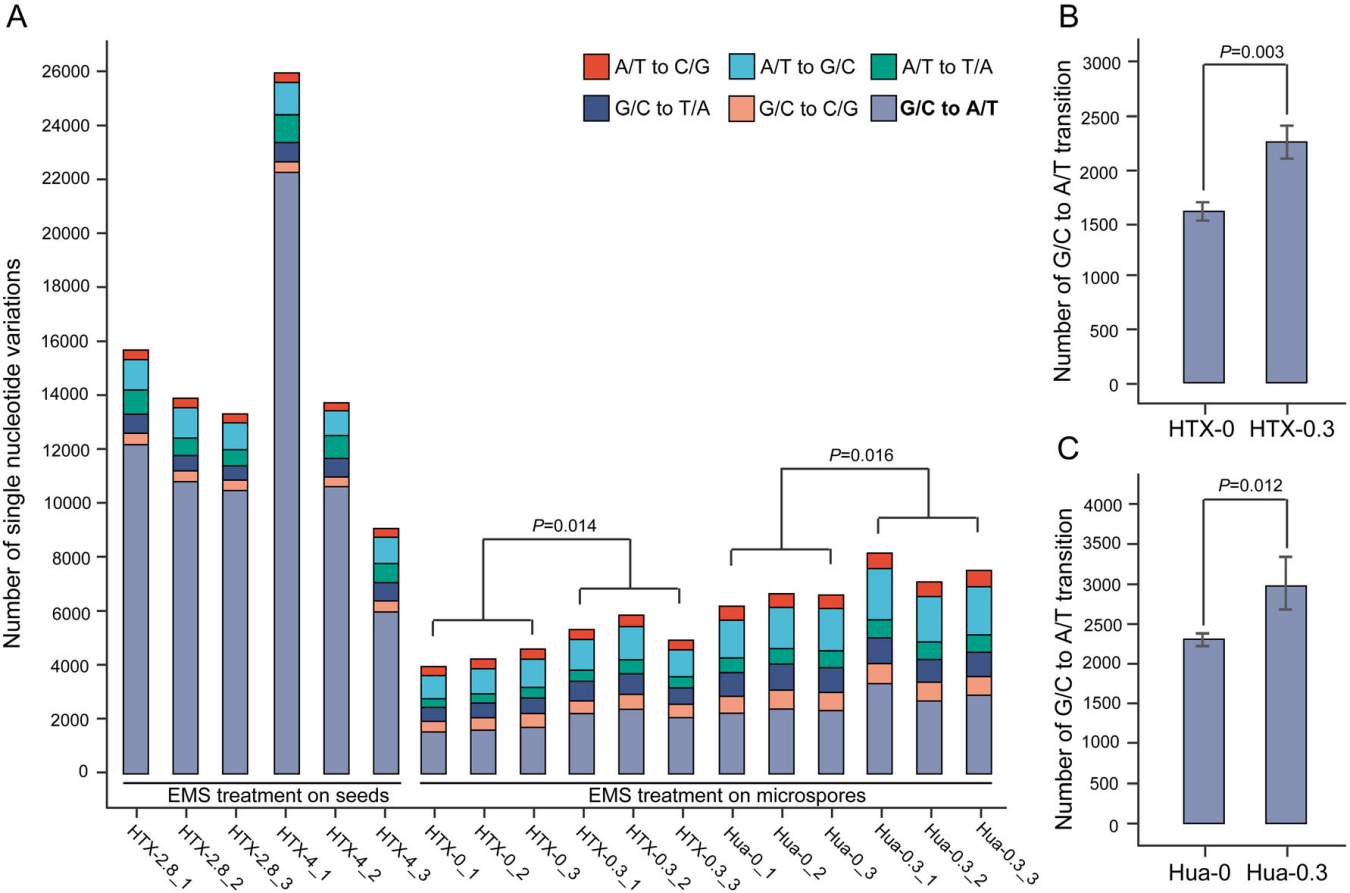
Single nucleotide transitions or transversions detected by whole-genome sequencing of mutated plants. **A** Overall number of single nucleotide differences detected in plants from mutagenized seeds (left, adapted from Jiang et al. 2022) or microspores (right). The G/C to A/T transition is a typical effect of EMS-induced mutagenesis, which significantly increased in regenerated plants treated with 300 mg/L EMS in both the HTX (**B**) and Hua30 (**C**) genotypes. The abbreviated name of each sample indicates the genotype, the EMS concentration, and the biological replicate. For example, HTX-2.8_1 indicates the first replicate of HTX treated with 2800 mg/L EMS

In addition, we counted the numbers of nucleotide transitions (A/T to G/C; G/C to A/T) or transversions (A/T to C/G; A/T to T/A; G/C to T/A; G/C to C/G) (Fig. 4A). The G/C to A/T transition, a typical effect of EMS-induced mutation, represented the majority of mutations in HTX mutants derived from EMS treatment of seeds in a previous study (Fig. 4A). Following treatment with 300 mg/L EMS in the induction medium, the ratio of G/C to A/T transition significantly increased in both Hua30 (*P* < 0.05) and HTX (*P* < 0.005) DH mutants (Fig. 4B and C), while the number of other types of mutations also significantly increased (*P* < 0.05). Non-G/C to A/T mutations were detected at a ratio over 50% in the regenerated plants following microspore culture both with and without EMS treatment.

Collectively, these results demonstrate that EMS-induced mutagenesis of isolated microspore cultures can produce homozygous mutations in the regenerated DH plants.

## Discussion

In this study, we developed a homozygous mutant population of cultivated barley after only one generation of seed multiplication by combining microspore culture with EMS-induced mutagenesis. We performed microspore mutagenesis in two distinct genotypes with a series of EMS concentrations together with whole genome re-sequencing of the regenerated plants, revealing the genotype- and EMS dosage-dependent effects on the regeneration efficiency of mutagenized DH lines.

Most mutant plant populations developed to date were obtained by mutagenesis of seeds (Irshad et al. 2020). Seeds before and after mutagen treatment are designated as M_0_ and M_1_ seeds, respectively (Caldwell et al. 2004). M_1_ plants that germinate from M_1_ seeds are usually heterozygous at each mutagenized locus. M_2_ plants and stocks of M_3_ seeds are normally used to construct mutant populations. For either breeding or genetic research, several rounds of self-pollination are required to obtain homozygous mutant seeds with stabilized phenotypic alterations. However, lethal or sterile effects can still occur in M_3_ plants or later generations (Jiang et al. 2022). In vitro culture of haploid microspores is a common approach for developing DH lines, producing regenerated plants homozygous at every genetic locus (Lu et al. 2008). Previous studies have included chemical mutagens in the microspore suspension or induction medium during microspore culture of *Brassica* species (Barro et al. 2001; Huang et al. 2016) and barley (Castillo et al. 2001; Gao et al. 2018). The regenerated M_1_ seedlings were homozygous, allowing their leaves to be sampled for DNA extraction to develop a TILLING population and their seeds (M_2_ seeds) to be directly sown for phenotyping in a field trial. In the current study, we obtained seeds from 1873 DH lines from the parental line Hua30 and 99 from HTX with nucleotide mutations. These DH lines with the same genetic background are valuable resources for phenotyping traits of interest.

Tissue culture-induced genomic variation (somaclonal variation) is a well-known phenomenon that has been widely studied (Bednarek et al. 2007; Zhang et al. 2014; Machczyńska et al. 2015; Bednarek et al. 2020). Previous studies have used amplification fragment length polymorphic (AFLP) markers to reveal the extent of somaclonal variations. In the present study, we utilized a new strategy to estimate gametoclonal variation. We subtracted the number of SNPs between plants obtained by seed germination vs. plants regenerated by microspore culture to calculate the genome-wide mutation frequency (1.84 and 1.23 mutations/Mb in Hua30 and HTX, respectively) that resulted from isolated microspore culture. Over 50% of the observed mutations differed from the G/C to A/T transition, the typical effect of EMS-induced mutagenesis. The mutation frequency, regeneration rate, and survival rate were shown to be genotype-dependent, as they were significantly higher in regenerated Hua30 vs. HTX plants with or without EMS treatment. For both genotypes, the mutation frequency was lower in the gene-coding regions than in the overall genome, implying that the non-coding regions have higher tolerance to mutations.

When we calculated the number of SNPs in the regenerated seedlings (0 vs. 300 mg/L EMS), we determined that ca. 0.35 mutations/Mb are induced by EMS genome-wide, accounting for 19.0% and 28.5% of that resulting from microspore culture without mutagenesis in Hua30 and HTX, respectively. Compared to the mutation frequency in the same genotype (HTX) obtained by mutagenesis of seeds with a much higher dosage of EMS (i.e., 2800 mg/L; 3.99 mutations/Mb), the mutation frequency of microspores obtained in this study was low, with only 1.57 mutations per Mb (300 mg/L EMS), i.e., 39.35% that of seed mutagenesis. The low mutation frequency in microspores treated with EMS is unexpected and is likely due to an insufficient dose of EMS. Indeed, microspores that are competent to undergo embryogenesis possess plasticity, and EMS treatment could impose stress and induce cell reprogramming (Testillano 2019). The totipotency of isolated microspores is a complicated program including dedifferentiation (embryogenic callus induction) and differentiation (plantlet regeneration). Although embryogenic calli were induced in the presence of EMS, a portion of the resulting mutations might have recovered or been removed by selection against mutations in essential genes during later development (i.e., plantlet regeneration). Therefore, more studies are needed to fully evaluate the efficiency of microspore mutagenesis.

We did not sequence regenerated plants from calli treated with a lower dosage of EMS, as a much lower mutation frequency would be expected in these lines. These DH lines with lower rates of mutation but homozygous mutations would be quite useful in future breeding programs or genetic studies due to minimized background noise. Enlarging the population size with saturated mutations, combined with a highly sensitive mutation detection method (i.e., Fast Identification of Nucleotide variants by droplet DigITal PCR [FIND-IT]) (Knudsen et al. 2022), may help identify sufficient numbers of mutants in target genes. Considering that the mutagen was included in the induction medium, followed by 19 days of culture, the main reason for the low mutation frequency is likely the low level of EMS utilized. As Hua30 microspores showed a certain level of tolerance to mutagenesis, a dosage of > 300 mg/L may be considered to increase the frequency of mutations in the barley genome.

## Data Availability

The whole genome re-sequencing clean reads datasets were deposited in NCBI database with the accession ID “SAMN32366957” to “SAMN32366969” under the project ID “PRJNA915006”.
